# Evaluation of the Impact of Comorbidities on Omadacycline Pharmacokinetics

**DOI:** 10.1128/aac.02397-21

**Published:** 2023-03-14

**Authors:** M. Trang, E. A. Lakota, M. C. Safir, S. M. Bhavnani, L. Friedrich, J. N. Steenbergen, P. C. McGovern, E. Tzanis, C. M. Rubino

**Affiliations:** a Institute for Clinical Pharmacodynamics, Inc., Schenectady, New York, USA; b Paratek Pharmaceuticals, Inc., King of Prussia, Pennsylvania, USA

**Keywords:** comorbidity, omadacycline, pharmacokinetics

## Abstract

Omadacycline is approved in the United States for the treatment of patients with community-acquired bacterial pneumonia or acute bacterial skin and skin structure infections. Analyses were undertaken to evaluate pharmacokinetic differences among subjects or patients stratified by comorbidities. Differences in clearance by smoking status, history of diabetes mellitus, chronic lung disease, hypertension, heart failure, or coronary artery disease were evaluated using a Welch two-sample *t* test. Smoking was the only significant comorbidity after correction for sex, with a clinically insignificant difference of 13%. Omadacycline dose adjustments based on these comorbidities do not appear to be warranted.

## INTRODUCTION

Omadacycline, which is a novel aminomethylcycline, received approval from the U.S. Food and Drug Administration for the treatment of patients with acute bacterial skin and skin structure infection (ABSSSI) or community-acquired bacterial pneumonia (CABP) in 2018 (1). As part of the drug development program, a population pharmacokinetic (PK) model was developed using data from multiple phase 1 and phase 3 studies (2). This model was a linear, three-compartment model with zero-order intravenous input and first-order absorption that used transit compartments to account for a delay in oral absorption. Results of the covariate analysis, which evaluated the influence of age, body size measures, renal function, the presence of cirrhosis, serum albumin concentrations, sex, race, and the presence of infection (various types) on the PK of omadacycline, demonstrated that sex was the only subject or patient characteristic that reduced interindividual variability to a statistically significant extent, with 15.6% lower clearance in females than in males. Thus, given the lack of covariates with a clinically meaningful impact on omadacycline exposure, dose adjustment on the basis of the covariates that were studied was deemed not to be warranted (1).

In the phase 3 OPTIC study, which was a study conducted to compare omadacycline- and moxifloxacin-treated patients with CABP (3), the data from which were included in the above-described population PK analysis (2), a mortality imbalance was noted. All-cause mortality rates of 2.1% and 1.0% were observed for omadacycline- and moxifloxacin-treated patients, respectively (3). The results of exploratory analyses failed to demonstrate differences between treatment groups to explain this mortality imbalance (4). As a result, two mortality risk scores, Pneumonia Patient Outcomes Research Team (PORT) risk class (5) and CURB-65 score (6), were evaluated as predictors of omadacycline PK, to ensure that omadacycline drug exposures were consistent across patients with different mortality risk. Results of this analysis demonstrated that differences in the mean area under the concentration-time curve (AUC) from 0 to 24 h were not statistically significant when stratified by PORT risk class (*P = *0.248) or CURB-65 score (*P = *0.745) (7). Given that the risk of death was not found to be associated with omadacycline exposure, these findings suggested that the observed mortality imbalance was not due to differences in omadacycline exposure and that dose adjustments based on these variables did not appear to be warranted. In an effort to further explore additional covariate effects, the above-described population PK model was used to evaluate differences in omadacycline PK among phase 1 subjects and phase 3 patients with ABSSSI or CABP stratified by comorbidities not previously evaluated.

Since AUC is derived from clearance and the ratio of the AUC to the MIC (AUC/MIC ratio) is the PK-pharmacodynamic index associated with efficacy for tetracyclines (8–11), clearance was the focus of these analyses. Using the above-described population PK model (2), individual *post hoc* clearance estimates were computed in NONMEM version 7.2 (ICON Development Solutions, Ellicott City, MD, USA) for 356 healthy subjects enrolled in 11 phase 1 studies, 16 subjects enrolled in a phase 1 renal impairment study, 30 subjects enrolled in a phase 1 hepatic impairment study, 31 patients enrolled in a phase 1b uncomplicated urinary tract infection study, 180 phase 3 patients with ABSSSI or CABP who were included in the original model development data set, and 202 phase 3 patients with ABSSSI who were included in the validation data sets (total *n *= 815). The comorbidities evaluated in this analysis were smoking status, history of diabetes mellitus, chronic lung disease (chronic obstructive pulmonary disease, asthma, emphysema, or chronic bronchitis), hypertension, heart failure, and coronary artery disease (CAD).

Baseline subject or patient descriptors of the PK analysis population, including stratification by comorbidity, are summarized in Table 1. Subjects or patients with a history of CAD, diabetes mellitus, heart failure, or hypertension were older, were heavier, had higher body mass index (BMI) values, and had lower creatinine clearance (CL_CR_) values, compared to those without a history of those comorbidities. No appreciable differences were seen between the subjects or patients who were current smokers and those who were nonsmokers or between those who had a history of lung disease and those without chronic lung disease.

**TABLE 1:** Demographic characteristics of the PK analysis population^*a*^

Variable	Data for:												
	All subjects or patients (*n* = 815)	Current smoker		History of chronic lung disease		History of CAD		History of diabetes mellitus		History of heart failure		History of hypertension	
		Yes (*n* = 191)	No (*n* = 512)	Yes (*n* = 43)	No (*n* = 718)	Yes (*n* = 22)	No (*n* = 693)	Yes (*n* = 24)	No (*n* = 691)	Yes (*n* = 12)	No (*n* = 703)	Yes (*n* = 94)	No (*n* = 721)
Age (mean ± SD) (yr)	39.8 ± 14.1	42.0 ± 11.2	40.6 ± 15.0	44.4 ± 14.9	40.4 ± 14.1	65.1 ± 13.3	40.0 ± 13.8	59.6 ± 11.5	40.2 ± 14.1	71.1 ± 13.5	40.3 ± 14.0	57.4 ± 12.4	37.5 ± 12.7
Weight (mean ± SD) (kg)	79.2 ± 15.6	79.8 ± 17.7	79.5 ± 15.5	78.9 ± 17.5	79.5 ± 15.8	82.0 ± 12.0	79.6 ± 16.1	87.0 ± 20.7	79.4 ± 15.8	81.8 ± 10.1	79.6 ± 16.1	88.6 ± 20.3	77.9 ± 14.4
Height (mean ± SD) (cm)	173 ± 9.19	174 ± 9.37	172 ± 9.22	170 ± 11.1	173 ± 9.12	168 ± 6.98	173 ± 9.32	170 ± 8.18	173 ± 9.32	167 ± 8.66	173 ± 9.28	170 ± 9.68	173 ± 9.07
Body surface area (mean ± SD) (m^2^)	1.92 ± 0.196	1.93 ± 0.207	1.92 ± 0.198	1.90 ± 0.229	1.93 ± 0.197	1.92 ± 0.156	1.93 ± 0.200	1.97 ± 0.224	1.92 ± 0.198	1.91 ± 0.133	1.93 ± 0.199	1.99 ± 0.244	1.91 ± 0.188
BMI (mean ± SD) (kg/m^2^)	26.5 ± 5.13	26.5 ± 6.35	26.9 ± 4.93	27.3 ± 5.88	26.6 ± 5.20	29.0 ± 3.99	26.6 ± 5.37	30.2 ± 7.08	26.6 ± 5.24	29.4 ± 4.32	26.7 ± 5.35	30.5 ± 6.30	26.0 ± 4.72
CL_CR_ (mean ± SD) (mL/min/1.73 m^2^)	101 ± 29.5	107 ± 31.8	97.1 ± 29.2	95.0 ± 38.3	101 ± 29.3	56.3 ± 31.4	102 ± 29.6	62.3 ± 29.7	102 ± 29.8	48.6 ± 26.5	102 ± 30.0	71.1 ± 35.6	105 ± 26.1
Serum albumin concentration (mean ± SD) (mg/dL)	4.25 ± 0.458	4.03 ± 0.420	4.36 ± 0.426	4.00 ± 0.442	4.28 ± 0.463	3.93 ± 0.648	4.22 ± 0.448	3.96 ± 0.629	4.22 ± 0.449	3.70 ± 0.494	4.22 ± 0.453	4.03 ± 0.530	4.28 ± 0.440
Sex (no./total no. [%])													
Male	574/815 (70.4)	139/191 (72.8)	347/512 (67.8)	21/43 (48.8)	510/718 (71.0)	14/22 (63.6)	480/693 (69.3)	12/24 (50.0)	482/691 (69.8)	5/12 (41.7)	489/703 (69.6)	58/94 (61.7)	516/721 (71.6)
Female	241/815 (29.6)	52/191 (27.2)	165/512 (32.2)	22/43 (51.2)	208/718 (29.0)	8/22 (36.4)	213/693 (30.7)	12/24 (50.0)	209/691 (30.2)	7/12 (58.3)	214/703 (30.4)	36/94 (38.3)	205/721 (28.4)

a For healthy volunteers enrolled in the 11 phase 1 studies, histories of chronic lung disease, CAD, diabetes mellitus, heart failure, and hypertension were assumed to be absent.

**FIG 1:**
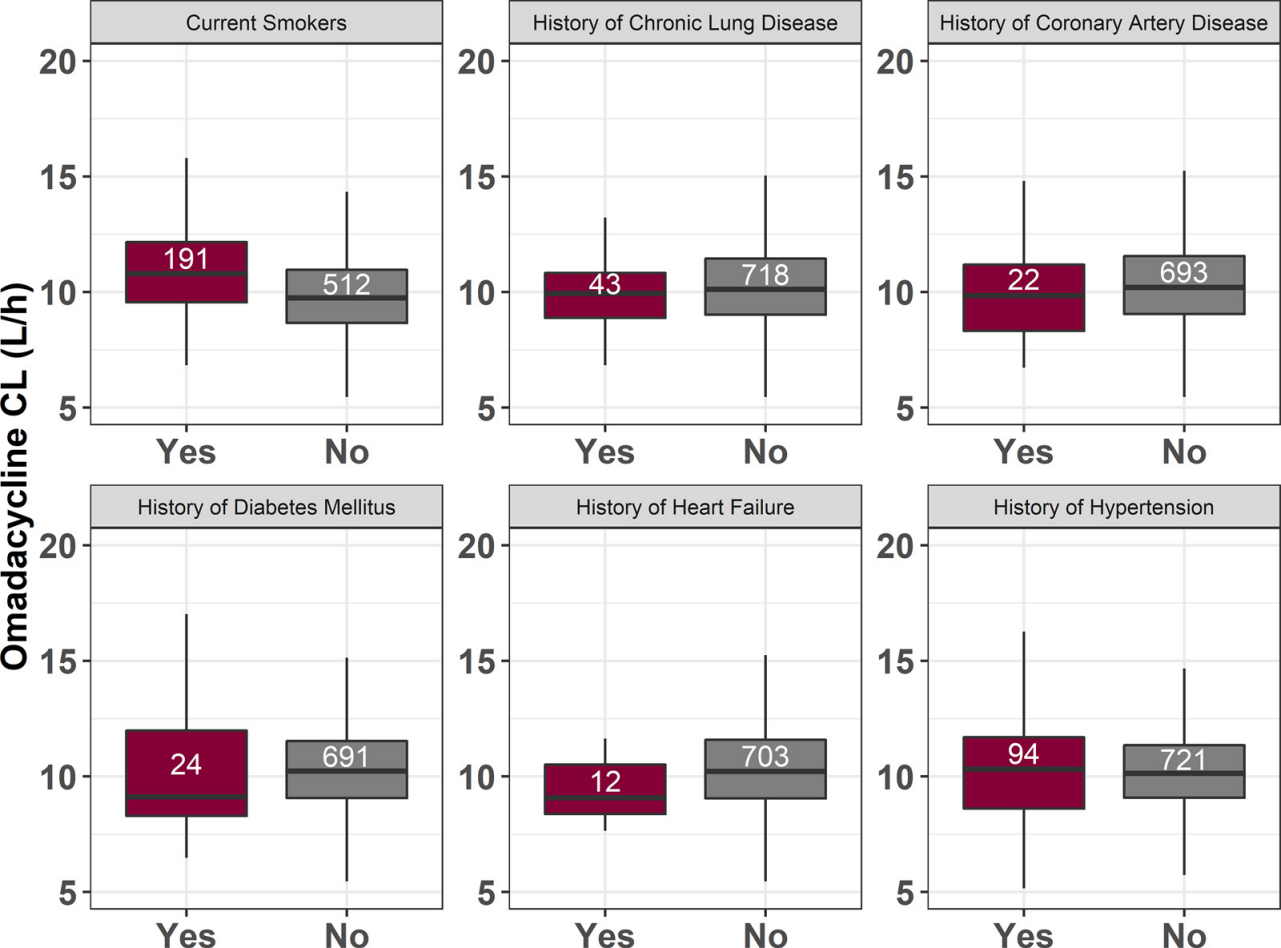
Box-and-whisker plots showing the distribution of omadacycline clearance (CL) values, stratified by various comorbidities. Counts for each group are provided in the respective boxes.

Differences in omadacycline clearance by the presence or absence of these comorbidities were evaluated using a Welch two-sample *t* test. Any potential imbalances in patient sex in the various groups were controlled for by using an analysis of variance (ANOVA) approach. Subjects or patients who were missing information for any covariates were removed from the respective analysis. Mean omadacycline clearances estimated by comorbidity are presented in Table 2. Distributions of omadacycline clearance values stratified by various comorbidities assessed are shown in Fig. 1. Mean clearance was statistically significantly higher in current smokers (current smokers, 11.2 L/h; nonsmokers, 9.97 L/h; *P < *0.001) and lower in subjects or patients with a history of heart failure (history of heart failure, 9.07 L/h; no history of heart failure, 10.4 L/h; *P = *0.0292). Mean omadacycline clearance was not significantly different based on a history of each of CAD (history of CAD, 9.89 L/h; no history of CAD, 10.4 L/h; *P = *0.235), diabetes mellitus (history of diabetes mellitus, 10.3 L/h; no history of diabetes mellitus, 10.4 L/h; *P = *0.888), chronic lung disease (history of chronic lung disease, 9.90 L/h; no history of chronic lung disease, 10.3 L/h; *P = *0.0737), or hypertension (history of hypertension, 10.4 L/h; no history of hypertension, 10.3 L/h; *P = *0.802).

**TABLE 2:** Mean omadacycline clearance estimates by the presence or absence of comorbidities

Comorbidity	Omadacycline clearance (L/h)	*P* ^*a*^
Current smoker (*n *= 703)		
Yes	11.2	<0.001
No	9.97	
History of chronic lung disease (*n *= 761)		
Yes	9.90	0.0737
No	10.3	
History of CAD (*n *= 715)		
Yes	9.89	0.235
No	10.4	
History of diabetes mellitus (*n *= 715)		
Yes	10.3	0.888
No	10.4	
History of heart failure (*n *= 715)		
Yes	9.07	0.0292
No	10.4	
History of hypertension (*n *= 815)		
Yes	10.4	0.802
No	10.3	

a Welch two-sample *t* test.

**FIG 2:**
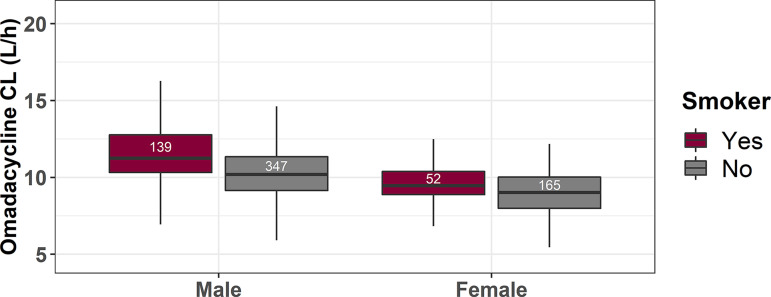
Box-and-whisker plot showing the distribution of omadacycline clearance (CL) values, stratified by smoking status and sex. Counts for each group are provided in the respective boxes.

Least-squares mean differences between groups after correction for patient sex are presented in Table 3. After correction for sex, smoking was the only comorbidity that remained statistically significant (*P < *0.001). Distributions of omadacycline clearance by sex and smoking status are provided in Fig. 2. A history of diabetes mellitus, chronic lung disease, CAD, or hypertension did not impact clearance. Although current smokers had statistically significant increases in omadacycline clearance, compared to nonsmokers, after accounting for the effect of sex, the least-squares mean difference of 1.13 represented an increase of 13% in the clearance of smokers. Given that a mean AUC of at least a halving or doubling is required to account for a full dilution change in MIC when the probability of achieving AUC/MIC ratio targets associated with efficacy is evaluated (12, 13), the magnitude of the difference observed between smokers and nonsmokers was not considered clinically relevant.

**TABLE 3:** Results of ANOVA comparisons of omadacycline clearance based on various comorbidities, with correction for sex

Comorbidity	LSM (90% CI)^*a*^	*P* ^*b*^
Current smoker	1.13 (0.804 to 1.45)	<0.001
History of chronic lung disease	−0.0572 (−0.669 to 0.554)	0.854
History of CAD	−0.434 (−1.29 to 0.419)	0.319
History of diabetes mellitus	0.280 (−0.541 to 1.10)	0.503
History of heart failure	−0.849 (−2.00 to 0.300)	0.147
History of hypertension	0.238 (−0.179 to 0.655)	0.263

a LSM, least-squares mean; CI, confidence interval.

b *t* statistic from ANOVA.

The results of previously-conducted covariate analyses for omadacycline, which included the evaluation of demographic variables and the assessment of subjects and patients, including those by indication, did not demonstrate clinically relevant differences in PK (2). In addition, results of analyses designed to assess PK differences for patients with CABP by mortality risk score did not demonstrate clinically relevant differences in exposure (7). To explore additional covariate effects, the analyses described herein were designed to assess PK differences for subjects or patients with CABP or ABSSSI stratified by additional comorbidities. A history of CAD, diabetes mellitus, chronic lung disease, or hypertension did not impact clearance. However, since the sample sizes for subjects or patients with these comorbidities were limited, these findings will need to be confirmed with additional studies. Although current smokers had statistically significant increases in omadacycline clearance, compared to nonsmokers, after accounting for the effect of sex, the difference was small and not considered clinically relevant. Thus, it is unlikely that the mortality differences noted in the phase 3 OPTIC study (3) were due to the systematic differences in the omadacycline exposure in specific subpopulations defined by the above-described comorbidities. We conclude that omadacycline dose adjustments based on the comorbidities assessed to date do not appear to be warranted.
